# Physical activity and chronic obstructive pulmonary disease: a scoping review

**DOI:** 10.1186/s12890-022-02099-4

**Published:** 2022-08-05

**Authors:** Xinyue Xiang, Lihua Huang, Yong Fang, Shasha Cai, Mingyue Zhang

**Affiliations:** Department of Nursing, The First Affiliated Hospital, College of Medicine, Zhejiang University, Hangzhou, Zhejiang Province China

**Keywords:** Physical activity, Chronic obstructive pulmonary disease, Barrier, Activity monitor, Scoping review

## Abstract

**Background:**

Reduced physical activity (PA) was the strongest predictor of all-cause mortality in patients with chronic obstructive pulmonary disease (COPD). This scoping review aimed to map the evidence on the current landscape of physical activity, barriers and facilitators, and assessment tools across COPD patients.

**Methods:**

Arksey and O’Malley’s scoping review methodology framework guided the conduct of this review. An electronic search was conducted on five English databases (PubMed, Cochrane Library, PsycINFO, CINAHL and Web of Science) and three Chinese databases (CNKI, CQVIP and WAN-FANG) in January 2022. Two authors independently screened the literature, extracted the studies characteristics.

**Results:**

The initial search yielded 4389 results, of which 1954 were duplicates. Of the remaining 135 articles, 42 studies met the inclusion criteria. Among the reviewed articles, there were 14 (33.3%) cross-sectional study, 9 (21.4%) cohort study, 4 (9.5%) longitudinal study, 3 qualitative study, 12 (28.7%) randomized control trials. The main barriers identified were older age, women, lung function, comorbidities, COPD symptoms (fear of breathlessness and injury, severe fatigue, anxiety and depression), GOLD stage, frequency of exacerbation, oxygen use, lack of motivation and environment-related (e.g., season and weather). Twelve studies have evaluated the effects of physical exercise (e.g., walking training, pulmonary rehabilitation (PR), pedometer, self-efficacy enhancing intervention and behavioral modification intervention) on PA and showed significant positive effects on the prognosis of patients. However, in real life it is difficult to maintain PA in people with COPD.

**Conclusions:**

Changing PA behavior in patients with COPD requires multidisciplinary collaboration. Future studies need to identify the best instruments to measure physical activity in clinical practice. Future studies should focus on the effects of different types, time and intensity of PA in people with COPD and conduct randomized, adequately-powered, controlled trials to evaluate the long-term effectiveness of behavioral change interventions in PA.

**Supplementary Information:**

The online version contains supplementary material available at 10.1186/s12890-022-02099-4.

## Introduction

Chronic obstructive pulmonary disease (COPD) is a common inflammatory lung disease characterized by persistent respiratory symptoms and airflow limitation [1, 2]. According to the World Health Organization (WHO), COPD is the third leading cause of mortality in the world [3]. The China Pulmonary Health (CPH) study showed that the overall prevalence of COPD was 8.6%, accounting for 99.9 million people with COPD in China [4]. For now, COPD has been a worldwide public health challenge to be paid attention to urgently.

Global Initiative for Chronic Obstructive Lung Disease (GOLD) guidelines recommend pharmacologic therapy, primarily inhaled corticosteroids and bronchodilators, as the preferred treatment for patients with stable COPD. However, these therapies do not effectively halt disease progression [1]. Due to the complexity of its pathophysiology, non-pharmacologic interventions (e.g., physical activity) can have significant effects in improving the quality of life and prognosis, with favorable socioeconomic benefits [5].

Physical activity (PA) is defined as any bodily movement produced by skeletal muscles that results in energy expenditure. Types of PA in daily life can be categorized into occupational sports, transportation (e.g., cycling and walking), household (e.g., yard work cleaning and home maintenance) or other activities [6]. Strong evidence demonstrates regular PA is beneficial to reducing the risk of many chronic diseases. Conversely, physical inactivity is a major risk factor for poor outcomes in people with COPD and also leads to early mortality death in patients harboring chronic diseases [7–9]. Due to activity-related breathlessness and decreased exercise tolerance, the majority of COPD patients are usually forced to reduce PA and adopt a sedentary lifestyle [10–12]. Specifically, the duration, intensity and counts of activity in people with COPD were reduced significantly [13]. In addition, PA levels in people with COPD began to decrease in the early stage of the disease and substantially declined over time [14–16]. As a result, the risk of readmission and mortality increased, and the patients’ quality of life fell [8, 17].

Fortunately, PA in people with COPD has gradually attracted the attention of scholars in recent years. The GOLD guidelines recommend regular physical activity for all patients with COPD, which significantly improves dyspnea, health status, and exercise tolerance [18]. Equally, both the American Thoracic Society (ATS)/the European Respiratory Society (ERS) note that PA can significantly improve health outcomes in people with COPD [19]. For example, a study found that COPD patients with high levels of physical activity had a 34% lower risk of 30-day readmission and a 47% lower risk of death within 12 months of discharge compared to inactive patients [20].

As for the barriers and facilitators of PA in people with COPD, a previous review found that the factors influencing the facilitators and barriers to PA following pulmonary rehabilitation included three themes, which were beliefs, social support, and the environment [21]. These findings also provide new insights into PA interventions for COPD patients in clinical practice, whereas it did not contain any quantitative findings. For PA interventions, a series of strategies currently implemented to treat low levels of PA in people with COPD, includes pulmonary rehabilitation, various types of exercise training, self-management, and behavior change strategies, reflect the complexity of this issue [22–25]. Another systematic review indicated that exercise training coupled with behavior change interventions (such as goal setting, motivational interviewing, and self-feedback) may be the optimal strategies to increase PA in people with COPD, but the specific type, time and intensity of PA are still unclear and need further research [26]. In addition, assessment of intensity of PA is important to ensure safety and the effectiveness of PA interventions in COPD. At present, two main PA assessment tools commonly utilized contain subjective assessment (questionnaire, diary, self-reported) and objective measurement (pedometer, accelerometer, activity monitor) [27–29]. However, the heterogeneity of measurement and reporting methods among different studies makes the results neither comparable nor easily synthesized.

For these reasons, a scoping review could be a better choice. It can quickly describe the research progress of a certain field, showing the scope, depth, breadth and deficiency, finally providing more information for the future. We consider incorporating qualitative and quantitative studies on this specific area from different perspectives, which may increase our understanding of complex physical activity behaviors. Therefore, this scoping review aimed: (1) to synthesize the evidence of barriers to PA in people with COPD; (2) to evaluate effectiveness of PA intervention in people with COPD; (3) to summarize the assessment methods of PA in people with COPD.

## Methods

The study was designed according to the reference framework developed by Arksey and O’Malley [30]. The main five stages were followed: (1) identify the research question, (2) identify relevant studies, (3) study selection, (4) charting the data, (5) summarize and report the results. This scoping review is reported according to the Preferred Reporting Items for Systematic Reviews and Meta-Analyses (PRISMA) Scoping Review guidelines [31] (see Additional file 1).

### Identify the research question

The main question of this review was: (1) What are the barriers to PA in people with COPD? (2) What are the interventions and outcomes of PA in people with COPD? (3) How to assess PA in people with COPD?

### Identify relevant studies

In this scoping review, we searched relevant publications for five English databases, including: PubMed, Cochrane Library, PsycINFO, CINAHL, Web of Science, and three Chinese databases, including: CNKI, CQVIP and WAN-FANG from their inceptions until January 2022. Boolean logic operators “AND, OR, NOT” were used for comprehensive retrieval. We searched for “chronic obstructive pulmonary diseases” and “physical activity”, all relevant keywords, mesh and other index terms, as well as combinations of these terms and appropriate synonyms, and these were used to construct the search strategy. Then we translated the English keywords into corresponding Chinese words for use in the Chinese databases. The search was restricted to human studies and papers in Chinese or English. The detailed search strategies can be found in additional file 1.

### Study selection

The inclusion criteria were the following: (1) the target population was people with COPD; (2) PA should be the major dependent or independent variables; (3) articles must be published in Chinese or English. The exclusion criteria were the following: (1) unpublished papers (including conference abstract, editorials, opinion papers, thesis); (2) full text conference abstracts that are unavailable; (3) non-human studies.

### Data extraction, synthesis, and charting

We used NoteExpressX9 for references management, classification, sorting, retrieval and editing. After the removal of duplicated records, two researchers independently screened the titles and abstracts of studies based on the following inclusion and exclusion criteria. Then, read the full text to determine the final references to be included in the study. In the case of disagreement, a third researcher participated in the discussion and made judgments. The final data was extracted from the included studies according to the following fields: (1) general information: author, country, publication date, (2) study characteristics: study designs, objectives, sample size (age and gender), findings, (3) physical activity assessment tools, (4) barriers associated with physical activity (see Additional file 1). Two reviewers worked on data analysis and synthesis. The included studies were described in the form of quantity and distribution, and the results were summarized by descriptive methods. Given the nature of scoping review, the risk of bias assessment is also not applicable.

## Results

### Articles retrieved

**Fig. 1:**
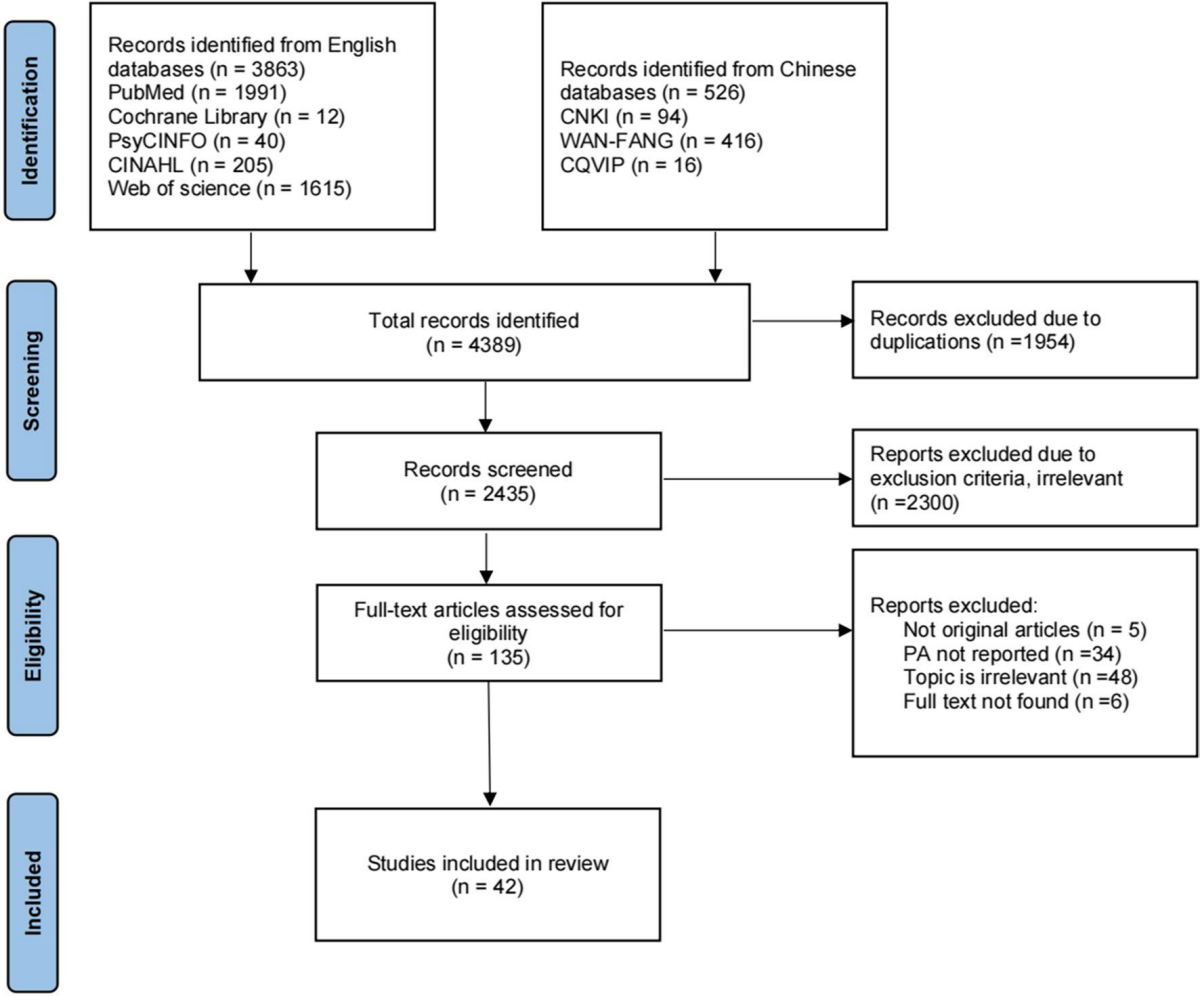
PRISMA flow diagram of study selection

All searches were carried out in January 2022. The initial search yielded 4,389 articles, with 526 records from Chinese databases and 3,863 records from English databases. After duplicates were removed, 2,435 articles were obtained for the screening process of the title and abstract. Finally, 135 full-text articles were analyzed for eligibility, and 42 articles were ultimately included [8, 9, 15, 32–70]. We described the searching process and outcome in Fig. 1.

### Article characteristics

**Fig. 2:**
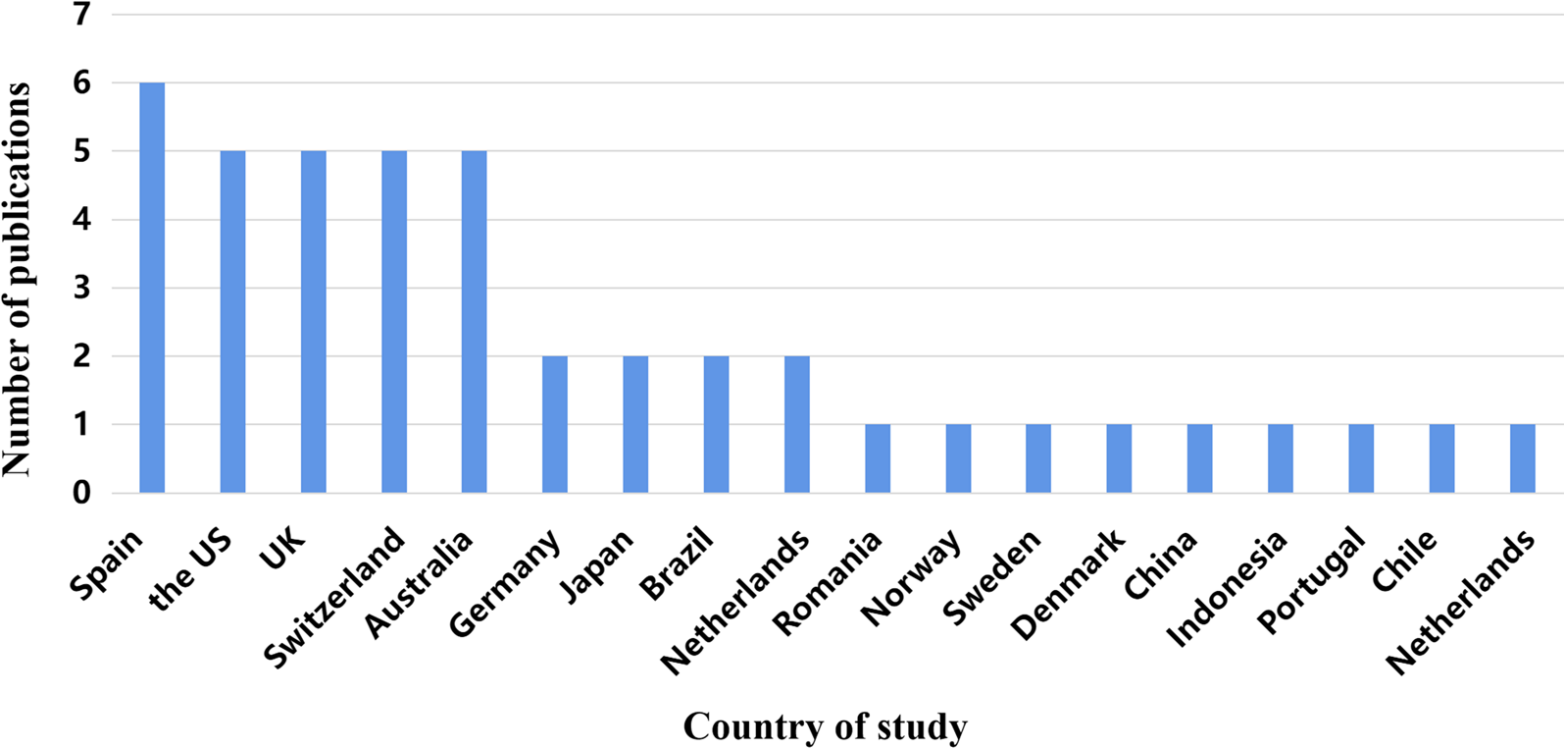
Number of studies per country

42 studies included in this review were mainly from 17 countries (Fig. 2). Spain was the most frequently studied country (6 studies, 14.3%) [8, 15, 40, 42, 53, 65], followed by the United States (5 studies, 11.9%) [33, 46, 62, 62, 69], the United Kingdom (5 studies, 11.9%) [35, 51, 57, 59, 60], Switzerland (5 studies, 11.9%) [48, 49, 54–56] and Australia (5 studies, 11.9%) [36, 47, 58, 63, 70]. The remaining 16 studies were conducted in 12 countries (Germany, Japan, Brazil, Netherlands, Romania, Norway, Sweden, Denmark, China, Indonesia, Portugal, Chile). Among the reviewed articles, study designs ranged from: n = 14 (33.3%) cross-sectional study [32–45]; n = 9 (21.4%) cohort study [8, 9, 46–52]; n = 4 (9.5%) longitudinal study [15, 53–55]; n = 3 (7.1%) qualitative study [56–58]; n = 12 (28.7%) randomized controlled trials (RCTs) [59–70]. Of these, 39 were quantitative studies and 3 were qualitative studies. The number of participants ranged from 21 to 483,603 in the quantitative studies and 18 to 28 in the qualitative studies. Mean age of participants across studies was above 40 years except one large cohort study [9]. The study showed that there were 32,535 patients had COPD, with aged 20–39 years (51.7%) and then 40–64 years (40.2%) [9]. Publication dates ranged from 2006 to 2021, and 27 (64.3%) studies were published in the period from 2017 to 2021 (see Table 1). A list of the 42 references, with all study characteristics, is presented in Additional file 1.

**Table 1:** General characteristics of included scoping reviews (n = 42)

Characteristic	Number of studies	Percentage (%)
*Publication year*		
2006–2010	2	4.8
2011–2016	12	30.9
2017–2021	27	64.3
Total	42	100.0
*Study designs*		
Cross-sectional study	14	33.3
Cohort study	9	21.4
Longitudinal study	4	9.5
Qualitative study	3	7.1
RCT	12	28.7
Total	42	100.0
*Methodology*		
Quantitative study	39	92.9
Qualitative study	3	7.1
Total	42	100.0

### Barriers of physical activity

Across all the 42 studies, 27 (64.3%) of studies summarized the barriers to PA in people with COPD, of which 14 used a cross-sectional design [9, 32–46], 4 used a longitudinal design [15, 53–55], 6 were cohort studies [47–52], and 3 used a qualitative design [56–58]. We categorized the barriers into four main aspects: sociodemographic variables, physiological factors, psychological factors and social environmental factors (see Table 2). For barriers, older age (6/27,22.22%), gender (3/27,11.11) education (2/27,7%) and race (1/27,4%) were the nonmodifiable sociodemographic variables; Fear of breathlessness (10/27,37.04%) were the main physiological factors; Lack of motivation (6/27,22.22%) and anxiety and depression symptoms (4/27,14.81%) were barriers from psychological factors; weather (4/27,14.81%) and lack of infrastructure (3/27,11.11%) were the social environmental factors.

**Table 2:** Barriers associated with physical activity (n = 27)

Categories	Barriers	Number of studies
Sociodemographic variables	Older age [32, 33, 46, 49, 57, 58]	6
	Gender [9, 33, 49]	3
	Employment status [34, 49]	2
	Race [32, 36]	2
	Lower educational levels [49]	1
	Smoking [43, 49]	2
Physiological factors	Lung function [9, 15, 33, 49, 55]	5
	Comorbidity [32, 38, 55, 58]	4
	Fear of breathlessness [15, 32, 34, 35, 38, 45, 50, 53, 54, 58]	10
	GOLD stage [34, 38]	2
	Frequency of exacerbation [33, 35, 38, 55, 56]	5
	Physical injury or illness [44, 45, 58]	2
	Oxygen use [37, 45]	2
	Fatigue [43, 49, 57]	3
	Worse Exercise capacity [36, 43, 49, 53, 58]	5
Psychological factors	Anxiety and depression symptoms [41, 42, 48, 49]	4
	Lack of motivation [32, 45, 49, 56–58]	6
	Underestimation of PA importance [36, 56, 57]	3
	Negative experiences of pulmonary rehabilitation [58]	1
Social environmental factors	Lack of family or friend support [46]	1
	Lack of infrastructure [40, 43, 44]	3
	Lack of willpower [43]	1
	Lack of time [44, 57]	2
	Social influence [40, 44]	2
	Weather [36, 51, 57, 58]	4
	Season [39, 45]	2
	Transport/finance [58]	1

Regarding the sociodemographic characteristics, studies focused on the effects of age and gender on PA in people with COPD. Low levels of PA are particularly important in women and older patients and it is related to worse functional and clinical characteristics [33]. Aging is associated with a decline in skeletal muscle mass and cardiopulmonary fitness, leading to impaired daily PA and maintaining independence [71]. In a study, the proportion of inactive people with COPD over 70 years old was significantly higher than other young age groups. Compared with men, women significantly reduced the intensity of walking and the amount of time they spent doing moderate and vigorous exercise [11]. Women begin to lose bone mass before menopause, and sedentary women as early as the 20 s [72]. Moreover, these differences are probably due to the socio-economic and cultural characteristics of the area, where few women are engaged in professional activities. Yet, they are used to staying home caring for the households and the family members.

In terms of the physiological factors, studies examined the effects of dyspnea, fatigue, comorbidity, and pulmonary function status on physical activity. Dyspnea is one of the most characteristic symptoms in people with COPD. Studies have shown that severe airflow obstruction and dyspnea are significantly associated with reduced levels of PA [15]. Patients with severe dyspnea often limit daily PA to alleviate dyspnea symptoms, ultimately creating a vicious cycle of decreased physical activity, muscle atrophy, and disease progression [15]. Fatigue is a highly prevalent symptom among people with COPD (71%) [73]. More patients with low PA had severe fatigue compared to the number of patients with moderate and high PA [43]. In a cross-sectional study, decreased cardiopulmonary function, systemic inflammation, and muscle weakness were strongly associated with PA in people with COPD [53, 74]. Chronic obstructive pulmonary disease is a systemic disease with multiple comorbidities, including cardiovascular disease, lung cancer, osteoporosis, diabetes, anxiety, and depression, and is associated with an increased risk of hospitalization and death [48, 75]. Inowe et al. [76] showed that in people with COPD combined with osteoporosis, decreased bone mineral density and bone damage increased the risk of fracture, which would further aggravate the disease, leading to decreased lung function and reduced PA level.

Of psychosocial factors, anxiety, depression and motivation were consistent positive correlates and determinants of PA in people with COPD. The prevalence of depression and anxiety is two to three times higher in people with chronic (long-term) medical conditions [77]. Anxiety and depressive symptoms are associated with poor exercise ability and quality of life. A multi-center study using objective assessment tools measured the relationship between anxiety and depressive symptoms and physical activity in 220 COPD patients at baseline and 6 months and 12 months of follow-up. Results had shown that symptoms of depression are prospectively associated with fewer steps per day and less time after 6 months of follow-up in people with COPD [42]. The lower motivation was a key barrier to PA. Fear of breathlessness may lead to low motivation and thereby reduce engagement in PA as patients are alarmed by a sensation of breathing difficulty that they are unable to manage [32]. In addition, one qualitative study based on grounded theory aimed to examine healthcare professionals’ perspectives on the importance and prescription of physical activity in people with COPD. The study found that although they acknowledged the importance of physical activity for people with COPD, there were few evidence-based strategies to enhance physical activity. The limitations include time constraints, treatment prioritization and perceived lack of expertise [78].

As for social-environmental correlates, weather conditions and seasonal variations may affect PA in people with COPD [36, 39, 45, 51, 57, 58]. Physical activity in a clean environment had greater health benefits than in more polluted areas. Alahmari et al. used a pedometer to examine the effects of the weather data (temperature, rainfall, sunshine) and environmental particulate matter on PA in people with COPD [51]. The results found that when air pollution was too hot or too cold, it caused obstacles to exercise and activity adherence, and patients' daily steps were significantly reduced. Stevens et al. investigated the relationship between environment and PA and self-rated health in people with COPD. Logistic and multivariate linear regression models showed a significant negative correlation between PA level and ozone pollution [79].

### Interventions for promoting physical activity

Twelve RCTs met the inclusion criteria reported that facilitators of PA among COPD patients [59–70]. The main interventions were related to physical exercise (e.g., walking training [63, 70], mobile health [60, 62], Pulmonary rehabilitation (PR) [59], PA counselling program [68], pedometers [64, 67], self-efficacy enhancing intervention [69], behavioral modification intervention (including education session, goal setting, motivational interview, identify potential barriers, self-monitoring, feedback, telephone calls) [61, 65, 66] (see Table 3). The majority of the reported physical activity programs had a total duration of 8–12 weeks. The longest intervention lasted about 12-months [65]. This study aimed to assess the 12-month efficacy and effectiveness of the Urban Training intervention on physical activity in people with COPD.

**Table 3:** Interventions associated with physical activity (n = 12)

Reference	Study design	Sample	Intervention	Outcome	Duration
Armstrong et al. [59]	RCT	48	PA behavioural modification interventions alongside PR	Primary outcome: PA (daily steps count) Secondary outcome: exercise capacity (6MWT); HRQOL (CAT, CCQ); anxiety and depression (HADS)	8 weeks
Bentley et al. [60]	RCT	30	SMART-COPD	Primary outcome: PA (daily steps count) Secondary outcome: Exercise capacity (ISWT); HRQOL (SGRQ, CAT); Anxiety and depression (PHQ-9); self-efficacy (Ex-SRES)	8 weeks
Larson et al. [61]	RCT	36	Active-life intervention (included walking, functional circuit training, and behavioral and educational strategies)	Primary outcome: PA (daily steps count) Secondary outcome: exercise capacity (6MWT)	10 weeks
Robinson et al. [62]	RCT	112	A web-based physical activity intervention + pedometer	Primary outcome: self-efficacy (Ex-SRES) Secondary outcome: PA (daily steps count); HRQOL (SF-36); exercise capacity (6MWT)	12 weeks
Wootton et al. [63]	RCT	101	Walk group (supervised, ground-based walking training, two or three times per week for 8–10 weeks)	Primary outcome: PA (daily steps count) Secondary outcome: exercise capacity (6MWT); HRQOL (SGRQ, CRQ); lung function (spirometry)	10 weeks
Widyastuti et al. [64]	RCT	40	Home pedometer assisted PA	Primary outcome: exercise capacity (6MWT) Secondary outcome: PA (daily steps count); HRQOL(CAT)	6 weeks
Etxarri et al. [65]	RCT	407	Urban training (a behavioural and community-based exercise intervention)	Primary outcome:PA (daily steps count) Secondary outcome: exercise capacity(6MWT); HRQOL (CAT, CCQ); anxiety and depression (HADS)	12 months
Cruz et al. [66]	RCT	32	A PA focused behavioural intervention	Primary outcome: PA (daily steps count) Secondary outcome: HRQOL (SGRQ)	12 weeks
Mendoza et al. [67]	RCT	97	Pedometers	Primary outcome: PA (daily steps count) Secondary outcome: exercise capacity(6MWT); dyspnoea (mMRC); HRQOL (SGRQ, CAT)	12 weeks
Altenburg et al. [68]	RCT	155	Physical activity counselling programme	Primary outcome: PA (daily steps count) Secondary outcome: exercise capacity (6MWT); HRQOL (CRQ, CCQ, SF-36); Anxiety and depression (HADS)	12 weeks
Larson et al. [69]	RCT	85	Self-efficacy and upper body resistance	Primary outcome: PA (daily steps count) Secondary outcome: Functional Performance Inventory	4 months
Breyer et al. [70]	RCT	60	Nordic walking	Primary outcome: PA (daily steps count) Secondary outcome: exercise capacity(6MWT); anxiety and depression (HADS); HRQOL (SF-36)	12 weeks

*PA* physical activity, *PR* pulmonary rehabilitation, *6MWT* six-minute walking test, *HRQOL* health-related quality of life health-related quality of life, *CAT* chronic obstructive pulmonary disease assessment test, *CCQ* clinical COPD questionnaire, *HADS* hospital anxiety and depression scale, *SMART* self-management supported by assistive, rehabilitative, and telehealth technologies, *ISWT* incremental shuttle walk test, *SGRQ* St. George’s respiratory questionnaire, *PHQ-9* patient health questionnaire, *Ex-SRES* exercise self-regulatory efficacy scale, *SF-36* short form 36, *CRQ* chronic respiratory questionnaire, *mMRC* modified medical research council scale

Walking is the most recommended form of aerobic exercises. An RCT study of “Nordic Walking” has shown that it is a simple, feasible and effective PA training program for patients with COPD. Nordic walking had a long-term positive impact on PA, dyspnea and other daily symptoms in people with COPD [70]. In another study, participants in the walking group received walking training sessions three times per week for 8 weeks, with significant improvements in health-related quality of life and exercise ability compared to usual care [63]. Pulmonary rehabilitation is the preferred non-drug treatment for COPD patients, which can improve the symptoms of dyspnea and exercise ability although the intervention did not maintain PA levels in the long term. Wshah et al. [80] embedded a 4-week behavior change program, consisting of a 30-min personalized face-to-face session and three follow-up visits, which aimed at reducing sedentary time, into pulmonary rehabilitation programs in people with COPD. PR combined with behavior change intervention can increase daily steps and reduce sedentary time compared to pulmonary rehabilitation alone, while its effect on sedentary behavior needs to be further explored. Several studies have shown that replacing 30 min of sleep or sitting times with 30 min of moderate to vigorous PA is consistently associated with improved lung function [81]. However, given that physical activity of moderate to vigorous intensity leads to an increase in ventilation and dyspnea, which serves as a barrier to engagement in regular physical activity. In contrast, light physical activity is more acceptable for patients that can promote long-term maintenance of physical activity, improve health-related quality of life and functional ability [36, 82]. Moreover, wearable activity monitors such as pedometers or accelerometers can help patients set goals, make plans, and self-monitor daily steps, exercise time and intensity, thus effectively improving patients’ exercise compliance. Qiu et al. [83] study showed that the use of walking counter significantly increased the daily steps and physical activity level of patients, yet it may not improve the physical activity or exercise ability of patients with moderate and severe COPD, and its long-term effectiveness has not been examined. Larson et al. conducted a 10-week “Active for Life with COPD” program in 36 COPD patients, including walking, functional circuit training, behavioral intervention and health education. Participants engaged in at least 60 min of daily activity (including 20 min of walking, 30 min of moderate-intensity exercise, and 10–15 min of stretching). The results showed that the behavior change program was feasible and acceptable, and people with COPD increased mean time spent standing/stepping by 36 min per day [61].

Among the twelve studies [59–70], PA was the primary outcome (10/12) [59–61, 63, 65–70]. The most common PA categories of variables used were daily steps count. The secondary outcome could be classified as exercise capacity (six-minute walking test (6MWT))(8/12) [59, 61–63, 65, 67, 68, 70]; health-related quality of life (HRQOL) Short Form 36 (SF-36) (3/12) [62, 68, 70], St. George’s Respiratory Questionnaire (SGRQ) (4/12) [60, 63, 66, 67], COPD Assessment Test (CAT) (5/12) [59, 60, 64, 65, 67], Clinical COPD Questionnaire (CCQ) (4/12) [59, 63, 65, 68]; anxiety and depression (Hospital Anxiety and Depression Scale (HADS)) (4/12) [59, 65, 68, 70] (see Table 3). All studies showed the effectiveness of PA interventions, including increased daily walking time, improved quality of life, and reduced risk of anxiety and depression. The frequency and intensity of training sessions varied between studies. Only four studies accounted for the time, intensity and frequency of PA in people with COPD, which recommends ≥ 30 min moderate physical activity ≥ 5 days per week [63, 65, 67, 70]. PA intensity was generally assessed using a dyspnea Borg scale score [70], maximum heart rate (HR) [70] and metabolic equivalents (METs) [63].

### Measurement of physical activity

Thirty-nine studies (92.3%) reported the measurements tools for assessing PA in people with COPD [9, 32–55, 59–70] (see Additional file 1). Of these, 11 (26.2%) utilized questionnaires including the International Physical Activity Questionnaire (IPAQ) [33, 43], Yale Physical Activity Survey (YPAS) [38], Physical Activity Scale for Elderly (PASE) [46], the Longitudinal Ageing Study Amsterdam Physical Activity Questionnaire (LAPAQ) [48, 49] and Clinical-PRO active Physical Activity [60, 65]. Three (7.1%) studies collected data using interview [8, 9, 47]. 27 (64.3%) studies utilized PA monitors, including pedometers (monitor daily step counts and provide real-time feedback) [46, 51, 54, 62, 64, 66–68] and accelerometers (measure the intensity of PA and provide energy expenditure) [15, 34–37, 39–42, 50, 52, 55, 60, 61, 63, 69, 70]. The instrument is a small and light device, which can be worn on the arm, waist, wrist, foot or thigh. Five types of accelerometers were most widely used, including the Sense Wear Armband (Body Media, Inc.), ActivPAL3 activity monitor, Actigraphy GT3X, DynaPort accelerometer, Actimarker EW4800 P-K. Compared with accelerometers, pedometers are cheaper and widely used in clinical practice. However, most studies failed to provide information on the validity of the instruments.

## Discussion

We performed a scoping review in this study to systematically map the barriers to physical activity, intervention strategies, and assessment tools in people with COPD. Of the 42 studies, there were 39 quantitative and 3 qualitative. Our findings indicate that people with COPD generally have low levels of PA. Compared to healthy, age-matched control subjects, people with COPD had a significant reduction in the duration, intensity and counts of PA. Fear of breathlessness, older age, and lack of motivation were the top three obstacles that prevent COPD patients from being more physically active. For PA interventions, our review discovered that regular PA was closely related to the health outcomes of patients, which can enhance patients' exercise ability, improve their quality of life, and delay disease progression. However, in real life it is difficult to maintain PA in people with COPD. Almost all studies reported methods for assessing PA in COPD, and 64.3% of studies used objective assessment tools.

PA is a complex behavior, which is affected by comprehensive factors such as sociodemographic variables, individual physiological, psychological, socio-cultural and environmental factors [9, 15, 32–46, 48–51, 53–58]. As the research mainly focuses on cross-sectional investigation, the influencing factors of the research design are relatively single, lacking comprehensive and systematic discussion. The cross-sectional study design is limited to speculating about the relationship between variables and has some limitations in exploring causality. Daily clinical symptoms such as fear of dyspnea, fatigue, anxiety, and depressive are associated with low levels of PA in people with COPD. Female patients showed lower energy expenditure in PA compared to male. This suggests that medical staff and caregivers of COPD patients should pay more attention to the PA of female patients [84]. According to the characteristics of middle-aged and elderly women, specific suggestions were put forward for activities, such as walking and square dancing. In addition, patients were encouraged to carry out activities with others, so as to enhance their sense of exercise self-efficacy and improve their PA level.

We found that people with COPD showed decreased levels of PA in the early stages of disease and deteriorated over time [9, 46–49]. Physical inactivity was the strongest predictor of all-cause mortality in people with COPD [50]. The findings in this review also indicate the importance to go beyond considering immutable sociodemographic factors to encompass factors related to COPD disease characteristics and behavioral changes. Early identification of symptoms and active intervention can help to delay disease progression and reduce mortality. A study found that over half (53.5%) patients were inactive. Patients with lower levels of PA are at higher risk for anxiety and depressive symptoms [48]. Among the COPD population, participants who were fully active had longer life expectancies than those inactive, an additional 2.4–4.0 years in men and 4.4–4.8 years in women [9]. Nonetheless, this study also has several limitations. Firstly, the sample size was small and the sex ratio of the study was uneven. In that case, it could not represent the whole population and may result in selection bias. Secondly, the study has different lengths of follow-up time and mainly focused on 1–3 years. Thirdly, most of the PA assessment tools were self-reported questionnaires, which may be affected by recall bias.

The results of 12 RCTs studies aiming at increasing PA in people with COPD showed that PA intervention strategies, including pulmonary rehabilitation, pedometers monitor, self-efficacy enhancement interventions and behavior change interventions, can enhance patients' exercise capacity and improve their quality of life [59–70]. As anticipated, a previous study showed that pulmonary rehabilitation can lead to changes in dyspnea and motor ability across COPD patients, but did not increase physical activity levels [85]. Compared with pulmonary rehabilitation alone, pulmonary rehabilitation combined with physical activity counseling and behavior change technology can effectively improve patients' physical activity levels [23]. A simple physical activity enhancement program using pedometers can effectively improve physical activity levels in people with COPD [68]. However, patients’ wearing compliance is poor. Pedometers cannot quantify patients' physical activity types and exercise-related environmental conditions, and its long-term effectiveness in PA needs to be further discussion. Theory-based behavior change interventions have been proved to be the most effective interventions [86]. The present behavioral modification interventions alongside pulmonary rehabilitation were well received by the vast majority of patients showing high compliance. However, such behavioral interventions may require a lot of health care resources as they are more time-consuming compared to PA tele-coaching. In addition, the timing, intensity, and frequency of PA remain obscure. Although guidelines recommend that all older adults should do at least 150 min of moderate-intensity physical activity a week, the majority of COPD patients fail to meet those guidelines [87]. Light-intensity physical activity has been shown to reduce all-cause and respiratory mortality in patients and is more readily accepted by patients. But it is not clear exactly what needs to be done to improve physical activity in the long term, which is what may be required for health benefits.

The quantification of PA level is very complex, and its accurate quantification may be a key step in the research and promotion of the PA. Accurate measurement of PA will further improve our understanding of the relationship between PA and health. The measurement of PA mainly included the frequency, duration, intensity and types of PA. At present, research shows that PA measurement tools can be divided into two main types: subjective assessment and objective measurement. Subjective assessment methods include questionnaire, self-report and interview [28]. The International Physical Activity Questionnaire is a widely recognized measurement method [88]. It mainly investigates the frequency and time of walking intensity physical activity and high-intensity physical activity during leisure time in the past 7 days, and calculates energy expenditure per unit body weight through metabolic equivalent. Although the questionnaire method has recall bias to some extent, it is widely used and more suitable for large epidemiological studies [28, 89]. Objective measurements, including pedometers and accelerometers, are mainly used to measure individual PA intensity and energy expenditure within a clinical trial [90]. Generally, to optimize the adherence of patients to wearing the device, the measurement should be worn at least 8 h during waking hours, except for bathing and swimming activities. Compared with traditional standard assessment tools, activity monitor has the advantages of high measurement accuracy and operability [27]. Moreover, it can improve patients' compliance and quality of life through real-time feedback. It’s a pity that the high price limits its application scope [91]. To ensure more scientific and objective research results, future research should combine subjective and objective measurement methods. Subjective methods such as activity log, questionnaire used to understand the characteristics and preferences of PA participants and activity monitors were used to monitor PA to obtain timely information and feedback, providing instrumental support for clinical staff to develop more scientific and perfect guidance measures.

## Strengths and limitations

The PRISMA was used to guide the reporting of the scoping review. Search terms were developed with the assistance of our team and the research librarian. The scoping review compiles information from studies with a wide range of designs and methodologies and is conducted with the rigor and transparency required. Importantly, this scoping review have gathered qualitative and quantitative data to identify barriers, intervention strategies, and assessment methods of PA in people with COPD, which will help researchers to understand PA behavior comprehensively and provide complementary data for PA intervention in clinical practice. Also, this scoping review has several limitations. We only searched the literature published in Chinese or English, thus omitting valuable data from relevant articles published in other languages. The quality evaluation of RCTs was not carried out in this review. The sample size was insufficient and could not represent the whole population. Therefore, in future research, We will expand our search to monitor the emergence of new literature and focus on literature published in other languages in this field to further understand the physical activity behavior of COPD patients.

## Conclusions

This scoping review identified 42 studies on the PA of people with COPD published from 2006 to 2021. Overall, these individuals have low physical activity levels. In particular, older and women had relatively lower levels of PA. PA is a complex behavior, which is difficult to be captured by simple measurement methods due to the influence of social population, physiology, psychology and environmental factors. Therefore, it is necessary to conduct effective intervention strategies. Pulmonary rehabilitation combined with physical activity counseling, self-efficacy enhancement and behavior change interventions were reported to have positive effects in enhancing COPD patients’ PA. However, its long-term effectiveness still needs to be further developed and validated in the future study. Sports program must be individualized, starting from low-intensity exercise. In addition, strengthening multidisciplinary cooperation and social support (from families, friends, and work) is utmost important to enhance the motivation of patients to exercise and promote changes in health behavior. More randomized controlled trials are recommended to evaluate the effects of different types, frequencies and intensities of physical activity on health-related quality of life in people with COPD.

## Supplementary Information

**Additional file 1.** Detailed and additional data of this manuscript.

## Data Availability

All data generated or analysed during this study are included in this published article [and its supplementary information files].

## Abbreviations

PA: Physical activity
COPD: Chronic obstructive pulmonary disease
PR: Pulmonary rehabilitation
GOLD: Global initiative for chronic obstructive lung disease
ATS: American Thoracic Society
ERS: The European Respiratory Society
RCT: Randomized controlled trials
HRQOL: Health-related quality of life health-related quality of life
6MWT: Six-minute walking test
HRQOL: Health-related quality of life
